# An Approach Based on Mammographic Imaging and Radiomics for Distinguishing Male Benign and Malignant Lesions: A Preliminary Study

**DOI:** 10.3389/fonc.2020.607235

**Published:** 2021-02-16

**Authors:** Yan Huang, Qin Xiao, Yiqun Sun, Zhe Wang, Qin Li, He Wang, Yajia Gu

**Affiliations:** ^1^ Department of Radiology, Fudan University Shanghai Cancer Center, Shanghai, China; ^2^ Department of Oncology, Shanghai Medical College, Fudan University, Shanghai, China; ^3^ Shanghai Center for Mathematical Sciences, Fudan University, Shanghai, China; ^4^ Institute of Science and Technology for Brain-Inspired Intelligence, Fudan University, Shanghai, China; ^5^ Human Phenome Institute, Fudan University, Shanghai, China; ^6^ Key Laboratory of Computational Neuroscience and Brain-Inspired Intelligence, Ministry of Education, Fudan University, Shanghai, China

**Keywords:** male breast lesions, mammography, radiomics, malignant, diagnosis

## Abstract

**Purpose:**

To develop and validate an imaging-radiomics model for the diagnosis of male benign and malignant breast lesions.

**Methods:**

Ninety male patients who underwent preoperative mammography from January 2011 to December 2018 were enrolled in this study (63 in the training cohort and 27 in the validation cohort). The region of interest was segmented into a mediolateral oblique view, and 104 radiomics features were extracted. The minimum redundancy and maximum relevance (mRMR) and the least absolute shrinkage and selection operator (LASSO) methods were used to exclude radiomics features to establish the radiomics score (rad-score). Mammographic features were evaluated by two radiologists. Univariate logistic regression was used to select for imaging features, and multivariate logistic regression was used to construct an imaging model. An imaging-radiomics model was eventually established, and a nomogram was developed based on the imaging-radiomics model. Area under the curve (AUC) and decision curve analysis (DCA) were applied to assess the clinical value.

**Results:**

The AUC based on the imaging model in the validation cohort was 0.760, the sensitivity was 0.750, and the specificity was 0.727. The AUC, sensitivity and specificity based on the radiomics in the validation cohort were 0.820, 0.750, and 0.867, respectively. The imaging-radiomics model was better than the imaging and radiomics models; the AUC, sensitivity, and specificity of the imaging-radiomics model in the validation cohort were 0.870, 0.824, and 0.900, respectively.

**Conclusion:**

The imaging-radiomics model created by the imaging characteristics and radiomics features exhibited a favorable discriminatory ability for male breast cancer.

## Introduction

The incidence of male breast cancer is approximately 1% of all breast cancers (1). However, the incidence of male breast cancer is increasing, and 2670 new cases of breast cancer were diagnosed in men in 2019, compared with 2500 cases diagnosed in 2018 (2, 3); research concentrated on imaging features in the diagnosis of male breast cancer is still limited. Male benign and malignant breast lesions vary in the clinical treatment and survival of patients; thus, it is vital to distinguish male benign and malignant breast lesions. Gynecomastia is the most common benign lesion in men, which could be bilateral or unilateral (4). When it appears unilateral, firm, and painless in palpation, it is difficult for clinicians to discriminate gynecomastia from breast cancer (5). Mammography was recommended in men aged 25 and older with questionable findings on physical examination by the American College of Radiology (6). Doyle (7) proposed that male breast cancer was usually appeared as an eccentric mass in the subareolar region in mammography. However, it is difficult for radiologists to discriminate benign and malignant breast lesions according to mammographic imaging to some degree, and this could be affected by radiologists’ experience in the process of discriminating benign and malignant breast lesions.

Radiomics, which extracts a large number of descriptive parameters from imaging data, is an emerging imaging postprocessing technology in radiology that can visualize more information from medical imaging (8). Radiomics has made great progress in many fields, and recent studies have explored the use of radiomics features in breast diseases, including distinguishing benign and malignant lesions (9), predicting the immunohistochemistry and status of Ki-67 (10, 11), calculating the state of sentinel lymph nodes (12), detecting the effect of neoadjuvant therapy and determining the risk of recurrence (13, 14), which were focused on female breast diseases; few existing studies have evaluated male benign or malignant breast lesions by radiomics. Hence, our study contributes to the investigation of a mammographic imaging-radiomics model for the diagnosis of male benign and malignant breast lesions.

## Material and Methods

### Patients

A total of 225 male patients who underwent mammography in our radiology department between January 2011 and December 2018 were evaluated retrospectively. The exclusion criteria were as follows: i) no pathology; ii) postoperative mammographic imaging; iii) poor imaging quality; iv) unilateral mammography examination. Ninety patients were enrolled in total (median age: 61 years, range 32 to 81) and were randomly classified into the training and validation cohorts by a computer algorithm at a ratio of 7:3.

### Mammographic Imaging Data Collection

**Figure 1:**
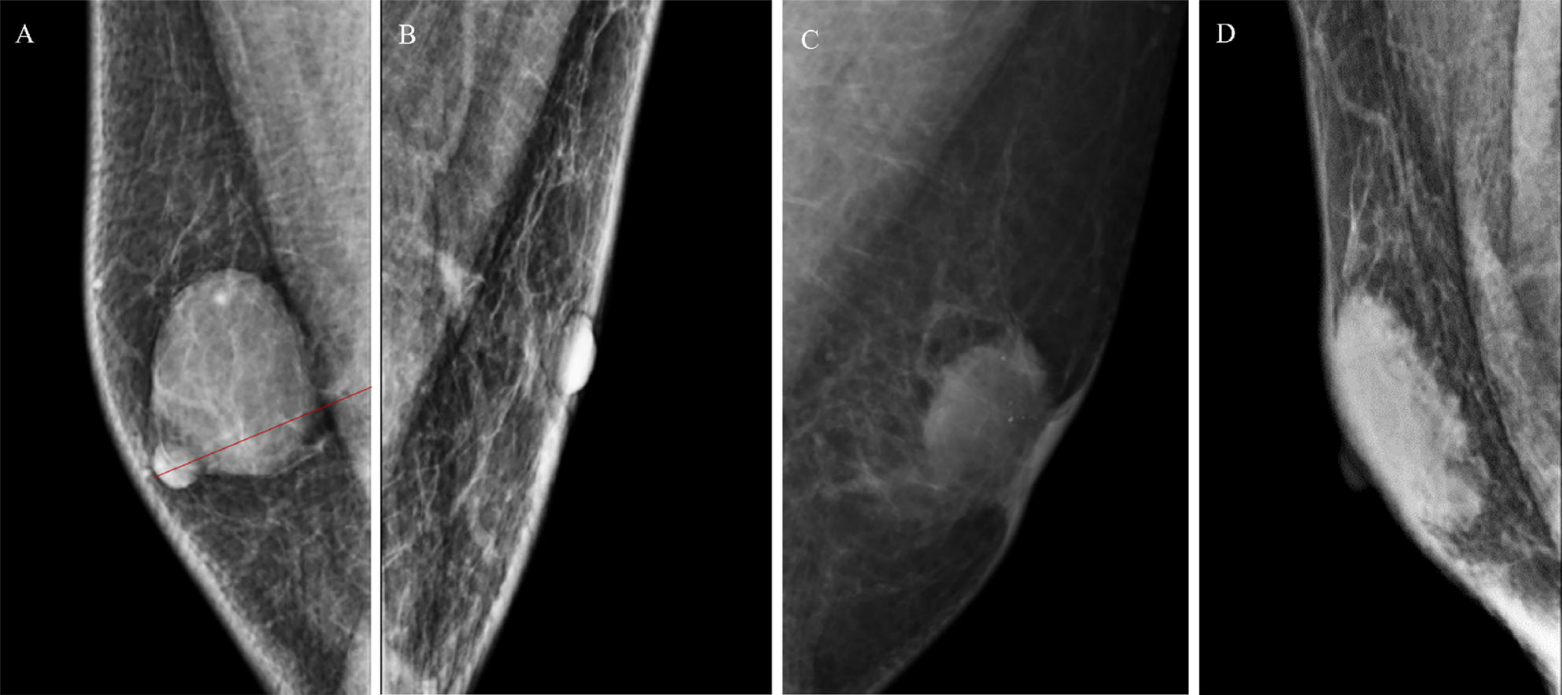
**(A)** A 63-year-old man with a mass in the right breast, located in the retro-areola area, eccentric lesion, pathology: encapsulated papillary carcinoma. **(B)** A 59-year-old man with a asymmetry in the left breast, located in the non-retro-areola area; pathology: invasive ductal carcinoma. **(C)** A 41-year-old man with a mass in the left breast, located in the retro-areola area, accompanying signs including calcification and skin thickening; pathology: invasive ductal carcinoma. **(D)** A 62-year-old man with a mass in the right breast, located in the retro-areola area; non-eccentric lesion; pathology: adenosis.

Mammography in the mediolateral oblique position was performed by Senographe DS (GE Healthcare, USA). Mammographic features (**Figure 1**) were reviewed and recorded as follows (15): i) lesion location: retro-areola, non-retro-areola. Lesions not clearly demarcated from the nipple were defined as retro-areola; otherwise, they were defined as non-retro-areola; ii) mammographic features: mass, asymmetry; iii) lesion density: the density of all lesions were recorded and classified as low density, isodensity and high density, according to a comparison with the pectoralis muscle; iv) lesion eccentricity: lesions evenly distributed around the perpendicular line (i.e., from the nipple to the pectoralis muscle) were defined as non-eccentric; otherwise, they were defined as eccentric; v) contralateral breast gland tissue: one study (16) reported that gynecomastia was bilateral in approximately half of patients. Thus, the contralateral breast gland tissue was enrolled in our study; vi) accompanying signs, including calcification and nipple, lymph and skin thickening. All imaging was evaluated by two radiologists who had 2–10 years of experience in breast imaging. When they had inconsistent decisions, an independent senior radiologist assessed the mammographic imaging. The agreement statistic was assessed between two radiologists. Continuous data were analyzed with intraclass correlation coefficients (ICCs), and categorical data were analyzed with Kappa coefficients. Variables (kappa values/ICCs >0.75) were further analyzed by univariate logistic regression analysis.

### Radiomics Feature Extraction

Mammographic imaging was normalized first. Then, regions of interest (ROIs) were segmented by manual methods using 3D Slicer software, which was completed by the same radiologists. In addition, 104 radiomics features were extracted in Python software, which included shape features, grey level cooccurrence matrix (GLCM), grey level size zone matrix (GLSZM), grey level run length matrix (GLRLM), neighboring grey tone difference matrix (NGTDM) and grey level dependence matrix (GLDM).

### Development of the Imaging-Only, Radiomics-Only, and Imaging-Radiomics Models

In the development of the traditional imaging model, all variables were used in a univariate logistic regression analysis to compare the differences between benign and malignant lesions. Variables (*p* < 0.1) were enrolled in the stepwise multivariate logistic regression analysis with Akaike information criterion employed as the stopping rule to build the imaging model. The diagnostic performance of the traditional imaging model was then validated in the validation cohort with the multivariate regression formula derived from the training cohort.

Next, the LASSO algorithm was used to exclude radiomics features to establish the radiomics score (rad-score) in the training cohort. Ten-fold cross-validation was implemented to avoid overfitting, and the rad-score was calculated for each patient *via* a linear combination of selected radiomics features that were weighted by their respective coefficients. The formula was applied in the validation cohort to calculate the corresponding rad-score.

Finally, mammographic imaging features in the imaging-only model and rad-score were used to build the imaging-radiomics model, and the process of validation was performed.

### Comparison of Models and Development of a Nomogram

Delong’s validation was used for the AUC between the mammographic imaging model and the imaging-radiomics model. We also calculated the probability of breast cancer for each patient with a logistic regression analysis and divided patients into the benign and malignant groups based on the probability of corresponding to the cut-off value with the highest Youden index. Compared with the actual breast cancer results, we calculated the sensitivity, specificity, accuracy, AUC, positive-predictive value (PPV), and negative-predictive value (NPV) for the three models in both the training and validation cohorts. Finally, a nomogram based on the most appropriate model was built. An internal validation of the nomogram was performed in the validation cohort.

### Development of Decision Curve Analyses

To evaluate the added value of the radiomics signature to mammographic imaging features in the diagnosis of male benign and malignant breast lesions, we developed two decision curves based on the imaging model and the imaging-radiomics model, and the clinical utility was demonstrated by calculating the net benefits for a range of threshold probabilities.

Statistical analysis was conducted with R software (version 3.6.2). A two-sided *p* < 0.05 was considered significant.

## Results

### Imaging Characteristics and Development of the Mammographic Imaging Model

Patient characteristics are shown in **Table 1**. There were no significant differences between the training and validation cohorts. Variables (kappa values/ICCs >0.75) were analyzed by univariate logistic regression analysis including age, mammographic types, lesion location, lesion density, lesion eccentricity, and accompanying signs (calcification, nipple retraction, and skin thickening, enlarged lymph nodes). In addition, univariate logistic regression demonstrated that year, mammographic types, lesion location, lesion eccentricity, and accompanying signs (calcification, nipple retraction, and skin thickening) were enrolled in a further analysis. After the multivariate analysis, lesion location, mammographic types, lesion eccentricity, accompanying signs (calcification and skin thickening) remained in the imaging-only model.

**Table 1:** Patient characteristics between the training and validation cohorts.

	Training (n=63)	Validation (n=27)	*p* value
Age	60(32~81)	61(38~79)	0.919
Mammographic types			0.693
Mass	49(77.8%)	22(81.5%)	
Asymmetry	14(22.2%)	5(18.5%)	
Contralateral breast gland tissue			0.203
yes	47(74.6%)	18(66.7%)	
no	16(25.4%)	9(33.3%)	
Lesion location			0.155
Retro-areola	42(66.7%)	22(81.5%)	
Non-retro-areola	21(33.3%)	5(18.5%)	
Lesion density			0.126
Isodense	24(38.1%)	15(55.6%)	
High	39(61.9%)	12(44.4%)	
Lesion eccentricity			0.962
Yes	40(63.5%)	16(59.3%)	
No	23(36.5%)	11(40.7%)	
Nipple retraction			0.349
Yes	15(23.8%)	9(33.3%)	
No	48(76.2%)	18(66.7%)	
Skin thickening			0.464
Yes	12(19.0%)	7(25.9%)	
No	51(81.0%)	20(74.1%)	
Enlarged lymph nodes			0.064
Yes	2(3.2%)	4(14.8%)	
No	61(96.8%)	23(85.2)	
Calcification			0.214
Yes	11(17.5%)	2(7.4%)	
No	52(82.5%)	25(92.6%)	

### Building a Radiomics Signature and the Diagnostic Validation

**Figure 2:**
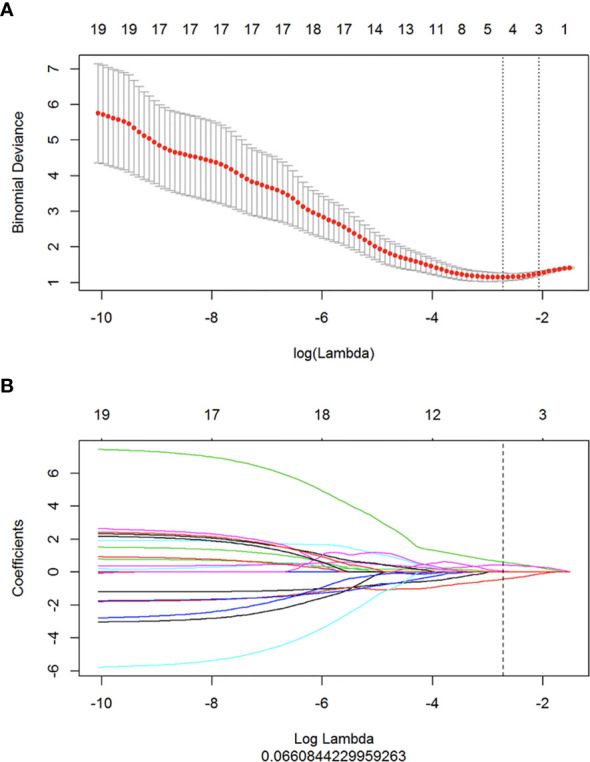
Radiomics feature selection by using mRMR and LASSO. **(A)** Tuning parameter (λ) selection in the LASSO model used ten-fold cross-validation *via* minimum criteria. The dotted vertical lines were drawn at the optimal values by using the minimum criteria and 1 standard error of the minimum criteria (the 1 + SE criteria). **(B)** The LASSO coefficient profiles of the 104 radiomics features. A coefficient profile plot was produced against the log (λ) sequence, and four features were chosen.

By using the LASSO regression model (**Figure 2**), 104 radiomics features were reduced to four potential predictors. These features presented in the rad-score were calculated by using the following formula: rad-score= 0.616*GLCM Maximal Correlation Coefficient+0.073*First-order 10 Percentile+-0.419*GLSZM Grey Level Non-Uniformity+0.419*First-order Mean + 0.249. In the training cohorts, the rad-score in benign and malignant lesions were 0.424 (range: -2.150 to 1.591) and 0.789 (range: -0.517 to 1.990), respectively. In the validation cohort, the rad-score in the benign and malignant lesions were 0.051 (range: -0.880 to 1.10) and 0.970 (range: -0.590 to 2.229), respectively. There was a significant difference in the rad-score between the benign and malignant groups in the training cohort (*p* < 0.001) using a univariate logistic regression analysis.

### Development of the Imaging-Radiomics Model

The stepwise logistic regression model selected the rad-score (OR: 15.622, 95% CI: 4.396~122.509), lesion location (OR: 13.107, 95% CI: 1.772~165.426), mammographic features (OR: 1.241, 95% CI: 0.115~15.146), lesion eccentricity (OR: 15.417, 95% CI: 1.883~241.890), accompanying signs, including calcification (OR: 30.562, 95% CI: 1.024~7418.389) and skin thickening (OR: 95.063, 95% CI: 5.220~5424.817), as predictors for breast cancer. Moreover, the rad-score was the dominant factor impacting the prediction of breast cancer in the imaging-radiomics model.

### Comparing Models and Determining the Nomogram Apparent Performance

**Figure 3:**
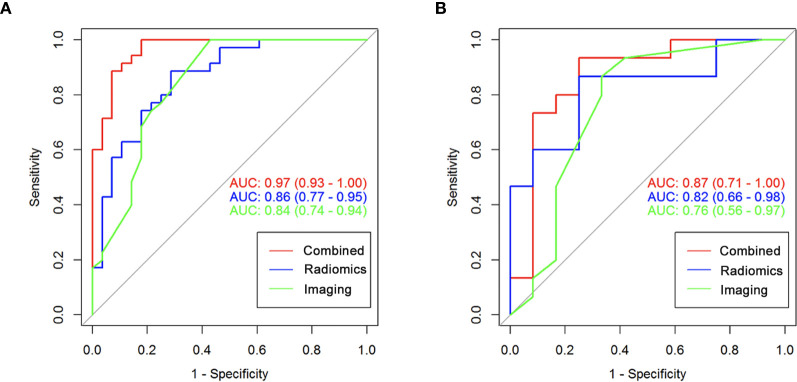
The ROC curve of imaging-only, radiomics-only and imaging-radiomics model in the training and validation cohorts. **(A)** The AUC of imaging-only, radiomics-only and imaging-radiomics model were 0.840, 0.860, and 0.970 in training cohort respectively. **(B)** The AUC of imaging-only, radiomics-only and imaging-radiomics model were 0.760, 0.820, and 0.870 in validation cohort respectively.

Delong’s validation was used to compare the AUC from the traditional imaging and imaging-radiomics models in the training and validation cohorts, respectively. As shown in **Table 2** and **Figure 3**, in the training cohort, the AUC in the imaging-radiomics model (AUC: 0.970; 95% CI: 0.930~1.000) was better than that in the imaging model (AUC: 0.840; 95% CI: 0.740~0.940), and there was a significant difference (*p* < 0.05). In the validation cohort, the AUC in the imaging-radiomics model (AUC: 0.870; 95% CI: 0.710~1.000) was higher than that in the imaging model (AUC: 0.760; 95% CI: 0.560~0.970), but the difference was not significant (*p >* 0.05).

**Table 2:** Diagnostic performance in the three models.

Model	AUC	95% CI	Accuracy	Sensitivity	Specificity	PPV	NPV
Imaging^a^	0.840	0.740~0.940	0.809	0.745	1.000	1.000	0.571
Imaging^b^	0.760	0.560~0.970	0.741	0.750	0.727	0.800	0.667
Radiomics^a^	0.860	0.770~0.950	0.810	0.714	0.886	0.833	0.795
Radiomics^b^	0.820	0.660~0.980	0.815	0.750	0.867	0.818	0.813
Imaging-radiomics^a^	0.970	0.930~1.000	0.921	0.875	1.000	1.000	0.821
Imaging-radiomics^b^	0.870	0.710~1.000	0.852	0.824	0.900	0.933	0.750

^a^training cohort; ^b^validation cohort.

**Figure 4:**
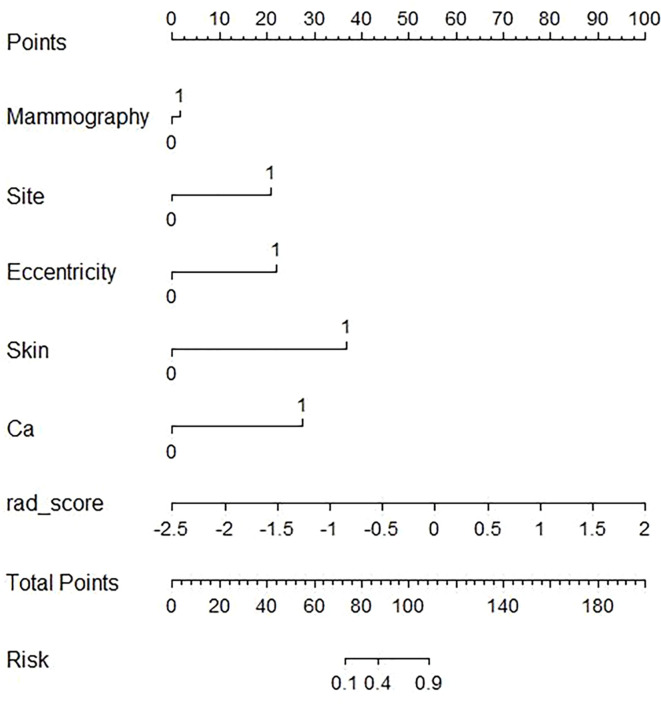
The developed imaging-radiomics nomogram for distinguishing male breast cancer. For mammography, zero represents a mass lesion, while one represents a asymmetry lesion. For the site, zero represents the re-areolar region and one represents the non-reareolar region. For eccentricity, skin (skin thickening), and Ca (calcification), zero represents not having those features, and one represents having those features. By calculating the scores of each point and locating it on the total score scale, the estimated probability of breast cancer can be assessed.

The imaging-radiomics nomogram was developed based on the imaging-radiomics model. As described in the nomogram (**Figure 4**), the rad-score accounted for the important proportion compared to the other imaging features, which made the radiomics signature the biomarker for male breast cancer.

### Clinical Use of the DCA

**Figure 5:**
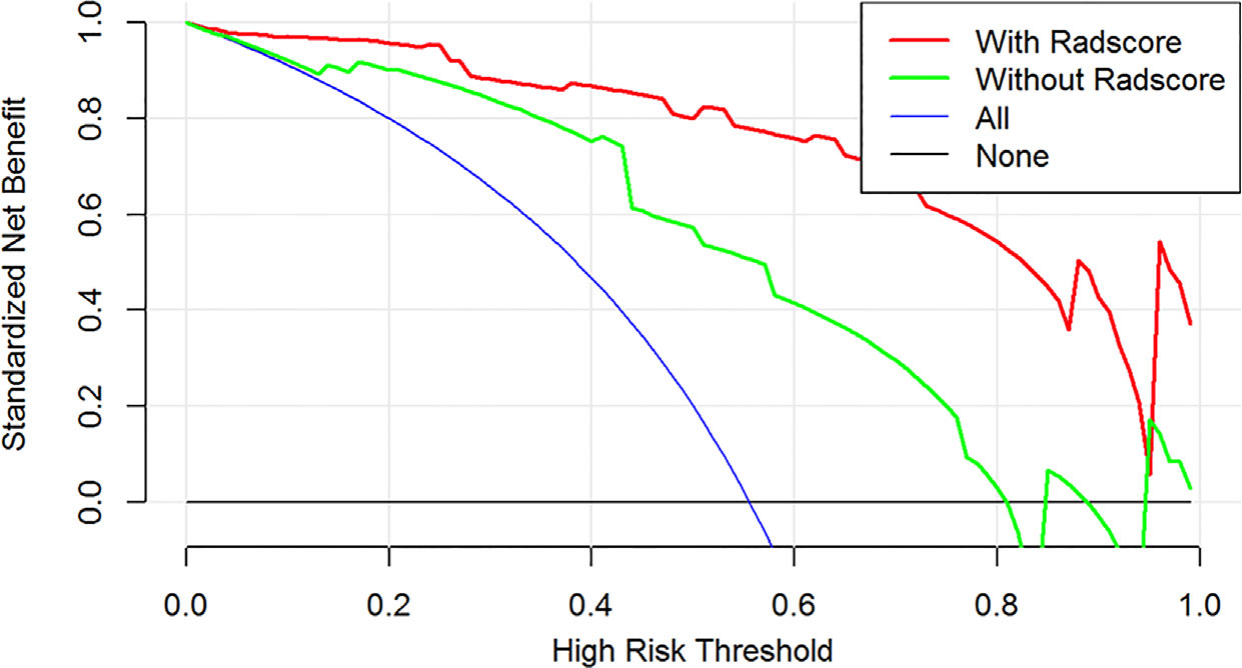
The DCA for the imaging and combined imaging-radiomics models. The y-axis is the net benefit. The green line represents the model of the imaging features, and the red line describes the combined imaging-radiomics features. The blue line represents the assumption that all patients have breast cancer. The black line represents the assumption that none of the patients have breast cancer. The threshold probability is the point at which a patient considers the benefit of treatment for high-risk breast cancer is equivalent to the harm of over-treatment for low-risk disease and thus reflects how the patient weighs the benefits and risks associated with the decision. The higher curve at any given threshold probability is the optimal prediction to maximize the net benefit. Across the various threshold probabilities, the imaging-radiomics curve shows a maximized net benefit compared with the traditional features for the individual performance.

The DCAs based on the imaging and imaging-radiomics models are shown in **Figure 5**. The imaging-radiomics nomogram achieved the most clinical utility with almost all of the threshold probabilities, which indicated that the imaging-radiomics model is a reliable tool to diagnose male benign and malignant breast lesions.

## Discussion

To the best of our knowledge, this is the first study to develop a model based on a radiomics analysis with traditional imaging features in mammography to distinguish male benign and malignant breast lesions. In addition, the imaging-radiomics model improved the performance compared with the imaging- and radiomics-only models, and the AUC values were 0.870, 0.760, and 0.820, respectively, in the validation cohort. Hence, we believe that imaging-radiomics would be useful to discriminate male malignant lesions and direct clinical decision making.

Five features, including asymmetry in mammography, lesions located in non-retro-areola region, lesion eccentricity, skin thickening and calcification, were used to differentiate male breast cancer in our study. Lesion types in mammography were classified as mass and asymmetry, and asymmetry lesions added to the risk of breast cancer. To date, several studies (17, 18) have suggested that lesion location and the relationship with the nipple have an important role in discriminating benign and malignant lesions; Doyle (7) suggested that male breast cancer was eccentric to the nipple and gynecomastia was central to the nipple. Therefore, based on these studies, both lesion and eccentricity were used in our study and remained after the multivariate logistic regression analysis. Moreover, lesions located in the non-retro-areola region and lesion eccentricity were associated with male breast cancer. In addition, skin thickening (17) is considered a malignant feature in male breast diseases, and further operations should be performed; this is consistent with our study. Moreover, previous studies (7, 19–21) suggest that calcification, including coarse and punctate calcifications, should be associated with malignancy in men and could be considered benign in females. Similarly, we found calcification to be a significant factor in male breast cancer.

We not only evaluated the imaging features but also explored more information in mammography images by radiomics analysis. Radiomics can extract various features from medical images and noninvasively determine tumor phenotypes (22, 23). In our study, the AUC values based on the rad-score were 0.860 and 0.820 in the training and validation cohorts, respectively. The diagnostic performance of the imaging-radiomics model improved, with AUC values of 0.970 and 0.870 in the training and validation cohorts, respectively. Some studies (9, 23, 24) utilized a radiomics analysis to distinguish female benign and malignant breast lesions, and they had a strong performance, with a maximum AUC of nearly 0.961. All these results indicate that radiomics has the potential to distinguish benign and malignant breast lesions.

Finally, we developed and validated an imaging-radiomics nomogram for clinicians to determine the breast cancer risk for every male patient. As the imaging-radiomics model had a higher AUC and more net benefits across the DCA, it may have a great potential to guide clinical treatment. We recommend that patients who are described as asymmetry, located in non-retro-areola, lesions eccentricity, skin thickening or calcification in the mammography and have a higher rad-score should undergo biopsy or surgery because these patients have a higher risk of breast cancer. We believe that the clinical use of this nomogram can not only be helpful to guide clinical decision but also reduce the burden of costs from surgery and anxiety associated with false-positive results.

Our study had certain limitations. First, this was a retrospective study, and selection bias could not be avoided. In addition, clinical features were not included in the model, and the performance of the model could be improved further. Finally, the 95% CI values of several features were broad in our study, and larger single- and multicenter clinical trials are needed to verify our results.

In conclusion, the imaging features and radiomics signature have the potential to discriminate male breast cancer. The imaging-radiomics nomogram represented in this study demonstrates the added value of the radiomics signature and may serve as a meaningful tool in the clinical management of male breast cancer.

## Data Availability Statement

The raw data supporting the conclusions of this article will be made available by the authors, without undue reservation.

## Author Contributions

YH and QX contributed equally to this work. All authors contributed to the article and approved the submitted version.

## Funding

National Natural Science Foundation of China (grant no. NSFC82071878); Clinical Research Plan of SHDC (grant no. SHDC2020CR2008A); Shanghai Anticancer Association FLIGHT PROJECT(grant no. SACA-AX-201903); Shanghai Science and Technology Foundation (grant no. 19DZ1930502); Sky imaging research fund of China international medical foundation (grant no. Z-2014-07-2003-06); Shanghai Natural Science Foundation (grant No. 20ZR1406400).

## Conflict of Interest

The authors declare that the research was conducted in the absence of any commercial or financial relationships that could be construed as a potential conflict of interest.
